# Home Health Value-Based Purchasing and Postacute Home Health Visits Among Older Adults With Dementia

**DOI:** 10.1001/jamanetworkopen.2026.12232

**Published:** 2026-05-13

**Authors:** Ming-Ting Yang, Aaron Bloschichak, Qiuyuan Qin, Helena Temkin-Greener, Shubing Cai

**Affiliations:** 1 Division of General Internal Medicine, Icahn School of Medicine at Mount Sinai, New York, New York; 2 Department of Public Health Sciences, School of Medicine and Dentistry, University of Rochester, Rochester, New York; 3 Department of Information Service, Nationwide Children’s Hospital, Columbus, Ohio

## Abstract

**Importance:**

Medicare home health is a critical postacute care source for individuals with Alzheimer disease and related dementias (ADRD). The Home Health Value-Based Purchasing (HHVBP) program has substantially reshaped financial incentives in home health, but its implications for patients with ADRD are unclear.

**Objective:**

To assess the association between residence in a state with an HHVBP program and home health service volume within 30 days of home health initiation among Medicare beneficiaries with ADRD, and how this varies by patient race and/or ethnicity, dual eligibility, and home health agency (HHA) racial composition.

**Design, Setting, and Participants:**

This cohort study linked 2021 to 2022 Medicare claims data with Home Health Focus and Provider of Services files for Medicare fee-for-service beneficiaries with ADRD older than 65 years who initiated home health care within 14 days of hospital discharge in the 9 states in which an HHVBP program was piloted compared with non-HHVBP states. The analysis was conducted from November 2024 to June 2025.

**Exposure:**

Residence in an HHVBP pilot state vs a non-HHVBP state.

**Main Outcomes and Measures:**

Number of nursing and therapy visits received within 30 days (main analysis) and 14 days (sensitivity analysis) of home health initiation. Negative binomial regression models with HHA random effects were estimated, and average marginal effects were reported.

**Results:**

The analytic cohort included 264 601 Medicare beneficiaries with ADRD (median [IQR] age, 83 [77-89] years; 160 947 males [60.8%]), of whom 68 765 (26.0%) resided in HHVBP states. The overall racial and ethnic composition included Asian (3.1%; n = 8099), non-Hispanic Black (8.9%; n = 23 634), Hispanic (6.3%; n = 16 562), and non-Hispanic White (81.7%; n = 216 306) individuals, as determined by Research Triangle Institute race codes from the Master Beneficiary Summary File. Adjusted 30-day outcomes showed HHVBP state residence vs non-HHVBP state residence was associated with 0.46 fewer nursing visits (3.94 vs 4.40 visits; average marginal effect [AME] = −0.46; 95% CI, −0.52 to −0.41; *P* < .001) and 0.32 more therapy visits (6.37 vs 6.05 visits; AME = 0.32; 95% CI, 0.24-0.39; *P* < .001). In non-HHVBP states, dual-eligible beneficiaries and those receiving care from HHAs primarily serving a racial and ethnic minority population received more nursing but fewer therapy visits compared with non–dual-eligible beneficiaries and those receiving care from HHAs serving a predominantly White population. These differences became smaller in HHVBP states. For example, therapy visit gaps between patients receiving care from HHAs primarily serving a racial and ethnic minority population and those receiving care from HHAs serving a predominantly White patient population narrowed from −0.81 (95% CI, −0.90 to −0.73) to −0.27 (95% CI, −0.44 to −0.09) visits in non-HHVBP vs HHVBP states (*P* < .001). The findings of increasing use of therapy visits and smaller group differences were consistent within a 14-day window.

**Conclusions and Relevance:**

In this cohort study of Medicare beneficiaries with ADRD, residence in an HHVBP state was associated with increased therapy use and smaller differences in home health service patterns between racial and/or ethnic minority and White patients, dual-eligible and non–dual-eligible beneficiaries, and patients receiving care from minority-serving HHAs and those receiving care from HHAs serving a predominantly White patient population compared with residence in a non-HHVBP state. These findings suggest that VBP models could reduce differences in postacute care.

## Key Points

**Question:**

What is the association between Home Health Value-Based Purchasing (HHVBP) programs and the volume of nursing and therapy services received within 30 days of home health initiation after hospital discharge among Medicare beneficiaries with Alzheimer disease and related dementias (ADRD)?

**Findings:**

In this cohort study of 264 601 Medicare beneficiaries with ADRD, residence in a state with an HHVBP program was associated with 0.46 fewer nursing visits and 0.32 more therapy visits.

**Meaning:**

These findings suggest that VBP models could reduce differences in postacute care.

## Teaser

This cohort study examines the association between Home Health Value-Based Purchasing programs and volume of nursing and therapy services after hospital discharge among Medicare beneficiaries with Alzheimer disease and related dementias.

## Introduction

Medicare home health is a key postacute care option providing homebound beneficiaries with skilled nursing and therapy services.^1^ Currently, more than 3 million Medicare beneficiaries use home health care, with approximately one-third of these home health episodes being for postacute care, making home health agencies (HHAs) the second most common discharge destination after hospitalization.^2,3^ Home health is particularly important for individuals with Alzheimer disease and related dementias (ADRD), who represent more than one-third of all home health users.^4^ This population typically requires more intensive and prolonged home health care and incurs substantially higher home health costs.^5,6,7^

Although home health is linked to improved patient outcomes for individuals with ADRD, such as higher rates of successful community discharge and lower institutional admissions,^8,9,10^ concerns remain about the quality and equity of care delivery. Prior studies have reported differences in access to high-quality HHAs and the frequency of services, particularly therapy visits, and patient outcomes by race, ethnicity, and socioeconomic status, raising concerns about equitable care for this patient population.^11,12,13,14,15^

To improve the quality and efficiency of care, the Centers for Medicare & Medicaid Services (CMS) implemented the Home Health Value-Based Purchasing (HHVBP) program in 2016. The pilot program, launched in 9 randomly selected states (Massachusetts, Maryland, North Carolina, Florida, Washington, Arizona, Iowa, Nebraska, and Tennessee), required all Medicare-certified HHAs in those states to participate. HHVBP applied performance-based payment adjustments based on quality metrics such as unplanned hospitalizations and successful community discharge.^16^ Preliminary evaluations reported a 7% improvement in quality scores and an estimated $190 million in annual Medicare savings.^17^ Based on these results, CMS expanded HHVBP nationwide in 2022. The year 2022 was the preimplementation year, during which CMS provided resources and training to HHAs without changes in reimbursement. The first performance year was 2023, and the first payment adjustments began in 2025.^18^ In addition to HHVBP, CMS introduced the Patient-Driven Groupings Model (PDGM) in January 2020, which fundamentally changed how HHAs are reimbursed. PDGM eliminated therapy visit thresholds and adopted a case mix–adjusted model based on diagnosis, functional impairment, and clinical complexity. The payment episode was also shortened from 60 to 30 days, with higher reimbursement rates for the initial 30-day period.^19^ Together, HHVBP and PDGM represent a substantial shift in HHA reimbursement, with potential implications for care delivery patterns, particularly the provision of skilled nursing and therapy services.

Although early evaluations of HHVBP demonstrated overall improvements in care quality, little is known about its implications for Medicare beneficiaries with ADRD, who have unique health needs. Recent literature suggests changes in care delivery patterns, such as visit type and frequency, may influence home health outcomes in this population.^20^ Therefore, it is important to understand whether HHVBP improves or exacerbates existing differences in home health visit type and frequency across various groups. Recent research has documented persistent racial and ethnic differences in the frequency of postacute home health services received by older adults with ADRD.^12^ However, it remains unclear whether service volume also varies by dual-eligibility status or the racial composition of HHAs.

This study examined the associations of the HHVBP program, implemented within the context of PDGM, with the volume and type of services delivered in the first 30 days of a home health episode among Medicare beneficiaries with ADRD. Specifically, we evaluated whether HHVBP is associated with changes in the frequency of nursing and therapy visits, and whether these changes vary by patient race, ethnicity, or dual-eligibility status or the racial composition of the HHA providing services.

## Methods

The University of Rochester Institutional Review Board approved the study and waived the informed consent requirement because data were deidentified. Reporting of results adhered to the Strengthening the Reporting of Observational Studies in Epidemiology (STROBE) guidelines.

### Data

This retrospective cohort study used multiple national datasets from 2021 to 2022, including the Medicare Provider Analysis and Review file, the Master Beneficiary Summary File (MBSF), Medicare Home Health Claims and Revenue files, the CMS Provider of Services files, Home Health Compare, Home Health Focus, Neighborhood Atlas data, and the USAFacts website.

The Medicare Provider Analysis and Review file contains information on hospitalizations, including admission and discharge dates and diagnosis-related group (DRG) codes. The DRG codes also enabled us to differentiate between medical and surgical hospitalizations and obtain the corresponding DRG weights. The MBSF contains sociodemographic and chronic condition data. The home health claims were used to identify beneficiaries who initiated home health visits after hospital discharge and the HHA provider number; the revenue files documented individual home health visit dates and types of services.

Next, we linked the individual-level data using the HHA provider number with CMS Provider of Services files, Home Health Compare, and Home Health Focus from Brown University.^21,22,23^ From these sources, we retrieved information on HHA characteristics, such as profit, branch operating status, and HHA quality. Lastly, we linked our datasets to the 2019 Area Deprivation Index from the Neighborhood Atlas, which provides information on community deprivation status,^24^ and to the USAFacts website to calculate monthly COVID-19 incidence.^25^ The analysis was conducted from November 2024 to June 2025.

### Cohort

We identified Medicare fee-for-service (FFS) beneficiaries aged 65 years or older with ADRD who had at least one hospitalization between 2021 and 2022. Beneficiaries with ADRD were identified using the ever indicator in the MBSF Chronic Conditions Segment. We included Medicare FFS beneficiaries who (1) were continuously enrolled in Medicare Parts A and B (without Medicare Advantage enrollment) for 6 months before and 1 month after the index hospitalization and (2) initiated home health care within 14 days of hospital discharge. This 14-day threshold was chosen based on prior literature.^26^ To focus on early postacute home health episodes, we excluded those who had received any home health services within 60 days before the start of the current home health care. Additionally, we excluded those with another hospitalization within 60 days before the index hospitalization to avoid capturing potential readmissions.

Of the remaining eligible hospitalizations, we randomly selected one hospitalization per beneficiary during the study period. We further excluded those with COVID-19 hospitalizations (which might have influenced home health care delivery), noncontiguous state residence, other race (due to small sample size), and missing covariates, resulting in a final cohort of 264 601 beneficiaries with ADRD. Beneficiaries excluded due to missing covariates represented approximately 0.1% of the cohort. Given this rate, selection bias is expected to be minimal. eTable 1 in Supplement 1 compares the baseline characteristics of included and excluded beneficiaries. The full cohort creation process is detailed in eFigure 1 in Supplement 1.

### Variables

The outcome variables were the number of nursing and therapy visits provided during the initial 30-day period of a home health episode. Visit counts were derived from the Home Health Revenue files by using home health revenue codes: nursing (055X) and therapy (sum of physical [042X], occupational [043X], and speech therapy [044X]).^27^ The 30-day window reflects the current payment period under the PDGM and captures a clinically vulnerable period.^19,28^

Key explanatory variables included HHVBP status, individual race and ethnicity, Medicare and Medicaid dual eligibility, and whether the beneficiary received care from a minority-serving HHA. HHVBP status was defined based on the beneficiary’s state of residence.^16^ Individual race and ethnicity were identified using the validated Research Triangle Institute–classified race variable from the MBSF (Asian, Black, Hispanic, or White).^29^ Dual eligibility was determined from the MBSF at the time of the index hospitalization. Minority-serving HHA status was defined based on the racial and ethnic composition of the agency’s patient population, using data from Home Health Focus.^23^ We calculated the percentage of beneficiaries from racial and ethnic minority groups for each HHA by subtracting the percentage of White recipients of home health care from 100%. Based on the distribution of this variable in our study data, HHAs were categorized as minority serving (coded as 1) if the percentage of beneficiaries from racial and ethnic minority groups was equal to or greater than 32%, representing the top 25% of HHAs in our sample; otherwise, they were categorized as serving a predominantly White population (coded as 0). HHAs with fewer than 11 home health enrollees were labeled as low-number-of-event (LNE) HHAs, a separate category for very small agencies (coded as 2).

We included a range of covariates at the individual, hospitalization, community, and HHA levels to control for potential confounding factors affecting the number of home health visits received. The selection of these covariates was informed by the prior literature.^8,10,30^ Individual-level characteristics included patient age, sex, presence of chronic conditions, and total Medicare cost within the 90 days preceding the index hospitalization. Characteristics of the index hospitalization included length of stay, intensive care unit (ICU) use, medical vs surgical hospitalization, and DRG weights (as a proxy for resource intensity). Community-level covariates included monthly newly diagnosed COVID-19 cases by beneficiaries’ county of residence and whether beneficiaries resided in a resource-deprived community.

At the HHA level, we accounted for organizational and quality characteristics that may influence service delivery patterns. These covariates included HHA profit status (for profit vs nonprofit), affiliation status (branch or not), geographic location, mean Quality of Patient Care star rating during the study period, and HHA size. Following the methodology used in the HHVBP program calculation, HHA size was specifically categorized based on annual patient volume: larger-volume HHAs were defined as those serving 60 or more unique Medicare beneficiaries, while smaller-volume HHAs fewer than 60 unique beneficiaries.^31^

### Statistical Analysis

We used negative binomial regression with HHA random effects to model 30-day nursing and therapy visit counts, adjusting for individual-, hospitalization-, community-, and HHA-level covariates. To examine whether the associations between explanatory variables and 30-day home health visits varied by HHVBP status, we included interaction terms between these variables and HHVBP state residence. The negative binomial model was chosen to account for the count nature of the outcome and address overdispersion identified in our analysis. Four models were estimated for each outcome: model 1 (unadjusted), model 2 (adjusted for individual-, hospitalization-, and community-level covariates), model 3 (further adjusted for HHA racial composition), and model 4 (fully adjusted, including other HHA characteristics). We calculated the adjusted counts and presented the average marginal effects (AMEs) to facilitate the interpretation of regression results. All statistical analyses were performed in SAS 9.4 (SAS Institute Inc) and Stata 18.0 (StataCorp LLC), with statistical significance set at a 2-sided α value of less than .05.

We conducted an additional analysis of nursing and therapy visits received during the 14 days of home health as a sensitivity analysis. This early period, often referred to as frontloading, has been found to be critical for home health recipients, and early visits have been associated with home health outcomes.^32,33^ Examining these visits allowed us to evaluate whether the observed visit patterns were consistent during the initial phase.

## Results

### Primary Analysis

The cohort comprised 264 601 Medicare beneficiaries with ADRD who had been hospitalized (median [IQR] age, 83 [77-89] years; 160 947 males [60.8%]), 68 765 (26.0%) of whom resided in HHVBP states (Table 1). During the initial 30-day home health period, patients received a median (IQR) of 4 (2-6) nursing visits and 6 (2-9) therapy visits. The overall racial and ethnic composition was Asian (3.1%; n = 8099), non-Hispanic Black (8.9%; n = 23 634), Hispanic (6.3%; n = 16 562), and non-Hispanic White (81.7%; n = 216 306). Overall, 16.2% of patients were dual-eligible, and 73.6% received care from White-dominant HHAs.

**Table 1.** Characteristics of the Study Cohort

Characteristic	No. (%)			*P* value
	Analytic cohort (N = 264 601)	Non-HHVBP (n = 195 836)	HHVBP (n = 68 765)	
Outcomes, median (IQR)				
No. of nursing visits	4 (2-6)	4 (2-6)	4 (2-6)	<.001
No. of therapy visits	5 (2-9)	5 (2-9)	6 (3-9)	<.001
Explanatory variables				
Race andethnicity				
Asian	8099 (3.1)	7128 (3.6)	971 (1.4)	<.001
Non-Hispanic Black	23 634 (8.9)	17 494 (8.9)	6140 (8.9)	
Hispanic	16 562 (6.3)	12 721 (6.5)	3841 (5.6)	
Non-Hispanic White	216 306 (81.7)	158 493 (80.9)	57 813 (84.1)	
Dual-eligibility status				
Nondual	221 654 (83.8)	161 612 (82.5)	60 042 (87.3)	<.001
Dual	42 947 (16.2)	34 224 (17.5)	8723 (12.7)	
HHA racial composition				
Predominantly White population	194 780 (73.6)	138 163 (70.6)	56 617 (82.3)	<.001
Minority population	64 645 (24.4)	53 657 (27.4)	10 988 (16.0)	
LNE	5176 (2.0)	4016 (2.0)	1160 (1.7)	
Individual-level covariates				
Age, median (IQR), years	83 (77-89)	83 (77-89)	83 (77-88)	.02
Sex				
Male	160 947 (60.8)	119 661 (61.1)	41 286 (60.0)	<.001
Female	103 654 (39.2)	76 175 (38.9)	27 479 (40.0)	
Total Medicare cost in preceding 90 d before index hospitalization, median (IQR), $	1305 (394-3393)	1279 (377-3371)	1379 (443-3447)	.18
Community-level covariates				
Resided in deprived community (ADI ≥85)				
No	251 229 (94.9)	184 255 (94.1)	66 974 (97.4)	<.001
Yes	13 372 (5.1)	11 581 (5.9)	1791 (2.6)	
County-level monthly newly diagnosed COVID-19 cases per 1000 older adults, median (IQR)	33 (14-66)	34 (15-68)	31 (13-62)	<.001
Hospitalization-level covariates				
DRG weights, median (IQR)	1.31 (0.94-1.87)	1.31 (0.94-1.87)	1.27 (0.93-1.87)	<.001
LOS, median (IQR), d	4 (2-6)	4 (3-6)	4 (2-6)	.89
ICU	73 252 (27.7)	54 576 (27.8)	18 776 (27.3)	.01
Hospitalization type				
Medical	219 066 (79.4)	155 330 (79.3)	54 736 (79.6)	.12
Surgical	54 535 (20.6)	40 506 (20.7)	14 029 (20.4)	
Chronic conditions				
AMI	44 708 (16.9)	33 253 (17.0)	11 455 (16.7)	.05
Asthma	59 812 (22.5)	44 408 (22.7)	15 404 (22.4)	.14
AF	116 654 (44.1)	85 866 (43.8)	30 788 (44.8)	<.001
Cancer	64 110 (24.2)	46 986 (24.0)	17 124 (24.9)	<.001
CKD	175 297 (66.2)	129 311 (66.0)	45 986 (66.9)	<.001
COPD	124 523 (47.1)	91 665 (46.8)	32 858 (47.8)	<.001
Depression	168 407 (63.6)	123 464 (63.0)	44 943 (65.4)	<.001
Diabetes	145 539 (55.0)	108 650 (55.5)	36 889 (53.6)	<.001
HF	156 552 (59.2)	117 089 (59.8)	39 463 (57.4)	<.001
Hip fracture	44 873 (17.0)	33 205 (17.0)	11 668 (17.0)	.94
HLP	246 581 (93.2)	182 176 (93.0)	64 405 (93.7)	<.001
HTN	256 880 (97.1)	190 062 (97.1)	66 818 (97.2)	.12
Ischemic heart disease	187 555 (70.9)	138 803 (70.9)	48 752 (70.9)	.92
Osteoporosis	96 510 (36.5)	71 598 (36.6)	24 912 (36.2)	.12
RA or OA	219 940 (83.1)	162 810 (83.1)	57 130 (83.1)	.74
Stroke	129 277 (48.9)	95 331 (48.7)	33 946 (49.4)	.002
HHA-level covariates				
5-Star rating				
Quality ≥3	216 980 (82.0)	158 508 (80.9)	58 472 (85.0)	<.001
Quality <3 or no star	47 621 (18.0)	37 328 (19.1)	10 293 (15.0)	
Location				
Rural	235 715 (89.1)	172 118 (87.9)	63 597 (92.5)	<.001
Urban	28 886 (10.9)	23 718 (12.1)	5168 (7.5)	
Profit status				
For-profit	163 913 (61.9)	119 635 (61.1)	44 278 (64.4)	<.001
Nonprofit	95 049 (35.9)	72 714 (37.1)	22 335 (32.5)	
Governmental	5639 (2.1)	3487 (1.8)	2152 (3.1)	
Operate branches				
No	158 709 (60.0)	118 631 (60.6)	40 078 (58.3)	<.001
Yes	105 892 (40.0)	77 205 (39.4)	26 687 (41.7)	
HHA size, total beneficiaries				
<60	6815 (2.6)	5283 (2.7)	1532 (2.2)	<.001
≥60	257 786 (97.4)	190 553 (97.3)	67 233 (97.8)	

Abbreviations: ADI, Area Deprivation Index; AF, atrial fibrillation; AMI, acute myocardial infarction; CKD, chronic kidney disease; COPD, chronic obstructive pulmonary disease; DRG, diagnosis-related group; HF, heart failure; HHA, home health agency; HHVBP, Home Health Value-Based Purchasing; HLP, hyperlipidemia; HTN, hypertension; ICU, intensive care unit; LOS, length of stay; LNE, low number of events; OA, osteoarthritis; RA, rheumatoid arthritis.

Table 2 reports the main results from a set of negative binomial regression models examining the association between HHVBP status and the frequency of 30-day home health nursing and therapy visits; the full results are reported in eTable 2 in Supplement 1. Residing in an HHVBP state vs a non-HHVBP state was associated with significantly fewer nursing visits (β = −0.10; 95% CI, −0.12 to −0.09; *P* < .001) and more therapy visits (β = 0.06; 95% CI, 0.05-0.07; *P* < .001) within the first 30 days of home health (model 1). These associations remained after adjusting for individual-, hospitalization-, community-, and HHA-level characteristics, with significance observed for individual race, ethnicity, and dual eligibility. Because the results for our main explanatory variables were consistent across models, we focused on interpreting the fully adjusted model (model 4).

**Table 2.** Results of Negative Binomial Regression Models^a^

Outcome	β (95% CI)							
	30-d Nursing visits^b^				30-d Therapy visits^b^			
	Model 1	Model 2	Model 3	Model 4	Model 1	Model 2	Model 3	Model 4
**Overall**								
No. of observations	264 601	264 601	264 601	264 601	264 601	264 601	264 601	264 601
No. of HHAs	7825	7825	7825	7825	7825	7825	7825	7825
HHVBP state residence (reference, non-HHVBP)	−0.10 (−0.12 to −0.09)^c^	−0.10 (−0.11 to −0.09)^c^	−0.05 (−0.07 to −0.04)^c^	−0.05 (−0.06 to −0.03)^c^	0.06 (0.05 to 0.07)^c^	0.06 (0.05 to 0.07)^c^	0.03 (0.02 to 0.04)^c^	0.01 (0.00 to 0.03)^d^
**Race and ethnicity (reference, non-Hispanic White) × HHVBP residence**								
Asian	−0.01 (−0.03 to 0.00)	−0.03 (−0.05 to −0.01)^c^	−0.04 (−0.06 to −0.02)^c^	−0.04 (−0.06 to −0.02)^c^	−0.03 (−0.05 to −0.01)^c^	−0.03 (−0.06 to −0.01)^c^	−0.01 (−0.03 to 0.01)	−0.01 (−0.03 to 0.01)
Asian × HHVBP	−0.12 (−0.17 to −0.07)^c^	−0.11 (−0.16 to −0.06)^c^	−0.09 (−0.14 to −0.04)^c^	−0.09 (−0.14 to −0.04)^c^	0.05 (0.00 to 0.11)^d^	0.05 (−0.00 to 0.10)	0.02 (−0.03 to 0.08)	0.02 (−0.03 to 0.08)
Black	0.02 (0.01 to 0.03)^c^	0.00 (−0.01 to 0.01)	−0.01 (−0.02 to 0.00)	−0.01 (−0.02 to 0.01)	−0.07 (−0.08 to −0.05)^c^	−0.03 (−0.04 to −0.02)^c^	−0.01 (−0.03 to −0.00)^d^	−0.02 (−0.03 to −0.00)^d^
Black × HHVBP	−0.01 (−0.04 to 0.01)	−0.01 (−0.03 to 0.01)	0.01 (−0.02 to 0.03)	0.01 (−0.02 to 0.03)	0.02 (−0.00 to 0.05)	0.02 (−0.01 to 0.04)	0.00 (−0.02 to 0.03)	0.00 (−0.02 to 0.03)
Hispanic	0.03 (0.02 to 0.05)^c^	0.02 (0.01 to 0.03)^c^	0.01 (−0.00 to 0.02)	0.01 (−0.00 to 0.02)	−0.07 (−0.09 to −0.06)^c^	−0.07 (−0.08 to −0.05)^c^	−0.04 (−0.06 to −0.02)^c^	−0.04 (−0.06 to −0.02)^c^
Hispanic × HHVBP	−0.03 (−0.06 to −0.00)^d^	−0.03 (−0.06 to −0.00)^d^	−0.01 (−0.03 to 0.02)	−0.01 (−0.03 to 0.02)	0.11 (0.08 to 0.14)^c^	0.10 (0.07 to 0.13)^c^	0.08 (0.05 to 0.11)^c^	0.07 (0.04 to 0.10)^c^
**Dual eligibility status (reference: non-dual status) **								
Dual	0.04 (0.04 to 0.05)^c^	0.03 (0.03 to 0.04)^c^	0.03 (0.02 to 0.04)^c^	0.03 (0.02 to 0.04)^c^	−0.13 (−0.14 to −0.12)^c^	−0.11 (−0.12 to −0.10)^c^	−0.10 (−0.11 to −0.09)^c^	−0.10 (−0.11 to −0.09)^c^
Dual eligibility × HHVBP	−0.03 (−0.05 to −0.01)^c^	−0.02 (−0.04 to −0.00)^d^	−0.02 (−0.04 to 0.00)	−0.02 (−0.04 to 0.00)	0.04 (0.02 to 0.06)^c^	0.04 (0.02 to 0.06)^c^	0.03 (0.01 to 0.05)^c^	0.03 (0.01 to 0.05)^c^
**HHA racial composition (reference: HHA serving predominantly White population × HHVBP residence)**								
Minority-serving HHA	NA	NA	0.11 (0.09 to 0.13)^c^	0.13 (0.11 to 0.15)^c^	NA	NA	−0.12 (−0.14 to −0.11)^c^	−0.14 (−0.15 to −0.12)^c^
Minority-serving HHA × HHVBP	NA	NA	−0.22 (−0.26 to −0.19)^c^	−0.23 (−0.27 to −0.20)^c^	NA	NA	0.08 (0.05 to 0.11)^c^	0.10 (0.07 to 0.13)^c^
LNE HHA	NA	NA	0.15 (0.12 to 0.19)^c^	0.12 (0.07 to 0.17)^c^	NA	NA	−0.35 (−0.38 to −0.31)^c^	−0.30 (−0.35 to −0.24)^c^
LNE HHA × HHVBP	NA	NA	−0.15 (−0.22 to −0.08)^c^	−0.16 (−0.23 to −0.09)^c^	NA	NA	0.29 (0.23 to 0.36)^c^	0.30 (0.23 to 0.37)^c^

Abbreviations: HHA, home health agency; HHVBP, home health value-based purchasing; LNE, low number of events; NA, not applicable; ×, interaction.

^a^ All models included HHA random effects to account for patient clustering.

^b^ Model 1 was an unadjusted model including only explanatory variables and their interaction terms. Model 2 further adjusted for individual characteristics (eg, age, sex, and chronic conditions), index hospitalization characteristics (eg, diagnosis-related group weights and length of stay), and community-level covariates (eg, residence in deprived community). Model 3 added HHA racial composition and interaction with HHVBP status. Model 4 was the fully adjusted model, including other HHA-level covariates.

^c^ *P* < .01.

^d^ *P* < .05.

In model 4, significant interaction terms were observed between HHVBP status and several variables of interest. Specifically, for nursing visits, significant interactions were identified for Asian race (β = −0.09; −0.14 to −0.04; *P* = .001), minority-serving HHA (β = −0.23; 95% CI, −0.27 to −0.20; *P* < .001), and LNE HHA (β = −0.16; 95% CI, −0.23 to −0.09; *P* < .001). We did not find significant interactions between Black race, Hispanic ethnicity, and HHVBP state residence. For therapy visits, significant interactions included Hispanic race (β = 0.07; 95% CI, 0.04-0.10; *P* < .001), dual eligibility (β = 0.03; 95% CI, 0.01-0.05; *P* = .008), minority-serving HHA (β = 0.10; 95% CI, 0.07-0.13; *P* < .001), and LNE HHA (β = 0.30; 95% CI, 0.22-0.37; *P* < .001). These significant interactions suggest that the association of explanatory variables with 30-day home health visits differed by HHVBP state residence.

As estimated coefficients in negative binomial models represent log counts of the expected outcome, we calculated and reported adjusted counts and AMEs (Table 3 and Figure; eFigure 2 in Supplement 1) to facilitate interpretation. Residents of HHVBP states received significantly fewer nursing visits (3.94 vs 4.40 visits; AME = −0.46; 95% CI, −0.52 to −0.41; *P* < .001) and more therapy visits (6.37 vs 6.05 visits; AME = 0.32; 95% CI, 0.24-0.39; *P* < .001) within the first 30 days than those in non-HHVBP states.

**Table 3.** AMEs of Explanatory Variables by HHVBP Status

Variable	AME (95% CI)^a^					
	30-d Nursing visits			30-d Therapy visits		
	Non-HHVBP	HHVBP	*P* value^b^	Non-HHVBP	HHVBP	*P* value^b^
Race or ethnicity (reference: non-Hispanic White)						
Asian	−0.17 (−0.25 to −0.09)^c^	−0.48 (−0.66 to −0.31)^c^	.001	−0.06 (−0.19 to 0.07)	0.09 (−0.22 to 0.39)	.40
Black	−0.02 (−0.07 to 0.03)	0.00 (−0.07 to 0.08)	.59	−0.11 (−0.18 to −0.02)^d^	−0.08 (−0.22 to 0.05)	.84
Hispanic	0.04 (−0.01 to 0.11)	0.02 (−0.08 to 0.12)	.63	−0.24 (−0.33 to −0.14)^c^	0.22 (0.04 to 0.39)^d^	<.001
Dual-eligibility status (reference: non-dual status)						
Dual	0.14 (0.10 to 0.18)^c^	0.06 (−0.01 to 0.12)	.04	−0.56 (−0.62 to −0.50)^c^	−0.42 (−0.53 to −0.30)^c^	.04
HHA racial composition (reference: predominantly White population						
Minority population	0.58 (0.50 to 0.66)^c^	−0.40 (−0.53 to −0.28)^c^	<.001	−0.81 (−0.90 to −0.73)^c^	−0.27 (−0.44 to −0.09)^c^	<.001
LNE	0.53 (0.29 to 0.76)^c^	−0.15 (−0.44 to 0.13)	<.001	−1.60 (−1.85 to −1.36)^c^	0.02 (−0.44 to 0.47)	<.001

Abbreviations: AME, average marginal effect; HHA, home health agency; HHVBP, Home Health Value-Based Purchasing; LNE, low number of events.

^a^ Estimated from the fully adjusted negative binomial regression model (model 4). Interpretation of the values is the adjusted differences in the 30-day nursing or therapy visits between groups and their counterparts. Any slight discrepancies between reported values and differences in adjusted counts are due to rounding.

^b^ *P* values were calculated using the pwcompare(effects) command in Stata syntax to test whether the 2 estimated AMEs were statistically significantly different.

^c^ *P* < .01.

^d^ *P* < .05

**Figure.**
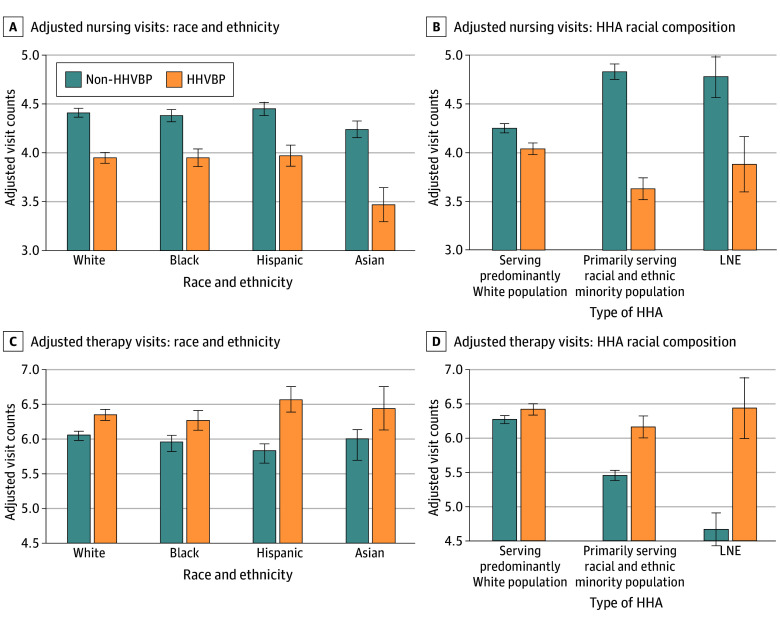
Bar Graphs of Adjusted Visit Counts of Explanatory Variables by HHVBP Status Values shown are adjusted counts estimated from the fully adjusted negative binomial regression model (model 4). HHA indicates home health agency; HHVBP, Home Health Value-Based Purchasing; LNE, low number of events.

Furthermore, race- and/or ethnicity-related and dual eligibility–related differences in home health visits varied by HHVBP status. In non-HHVBP states, dual-eligible beneficiaries received significantly more nursing visits than their non–dual-eligible counterparts (4.51 vs 4.38 visits; AME = 0.14; 95% CI, 0.10-0.18; *P* < .001; 3.0% more visits), as did those receiving care from minority-serving HHAs compared with those receiving care from HHAs serving a predominantly White patient population (4.83 vs 4.25 visits; AME = 0.58; 95% CI, 0.50-0.66; *P* < .001; 13.6% more visits). Conversely, significantly fewer therapy visits were received by Hispanic compared with non-Hispanic White beneficiaries (5.84 vs 6.07 visits; AME = −0.24; 95% CI, −0.33 to −0.14; *P* < .001; 3.8% fewer visits), dual-eligible compared with non–dual-eligible beneficiaries (5.58 vs 6.14 visits; AME = −0.56; 95% CI, −0.62 to −0.50; *P* < .001; 9.1% fewer visits), and those receiving care from minority-serving HHAs compared with those receiving care from HHAs serving a predominantly White population (5.46 vs 6.28 visits; AME = −0.81; 95% CI, −0.90 to −0.73; *P* < .001; 12.9% fewer visits).

These differences became smaller in HHVBP states. For example, the difference in the number of nursing visits between those receiving care from minority-serving compared with HHAs serving a predominantly White population was reversed, from 0.58 more visits in non-HHVBP states (4.83 vs 4.25 visits; 13.6% more visits) to 0.40 fewer visits in HHVBP states (3.63 vs 4.04 visits; 9.9% fewer visits; *P* < .001). For therapy visits, Hispanic beneficiaries received significantly more visits in HHVBP states compared with White individuals (AME = 0.22; 95% CI, 0.04-0.39; *P* < .001), reversing the direction observed in non-HHVBP states (Table 3). Similarly, the therapy visits gaps between those receiving care from minority-serving compared with HHAs serving a predominantly White population narrowed, improving from 0.81 fewer (95% CI, −0.90 to −0.73; *P* < .001) to 0.27 fewer visits (95% CI, −0.44 to −0.09; *P* = .002) in HHVBP states.

### Sensitivity Analysis

Results from the 14-day sensitivity analysis are reported in eTables 3 and 4 and eFigure 3 in Supplement 1. During this early period, no statistically significant differences were noted in nursing visits by HHVBP state residence (AME = 0.22; 95% CI, −0.14 to 0.06; *P* = .23). However, the findings for therapy visits remained consistent with the primary analysis, with individuals in HHVBP states receiving significantly more visits (AME = 0.18; 95% CI, 0.14-0.23; *P* < .001).

## Discussion

This study examined the association between HHVBP state residence and the frequency of 30-day home health visits among hospitalized older adults with ADRD in the context of recent home health PDGM payment reforms. Our findings revealed distinct postacute home health service patterns by HHVBP status: residents of HHVBP states received fewer nursing visits but more therapy visits within the first 30 days of home health. Furthermore, we identified associations of individual race, ethnicity, and dual eligibility and HHA racial composition with visit frequency that differed significantly by HHVBP status. The sensitivity analysis further supported the findings of more therapy visits in the first 14 days, while no differences in nursing visits were noted by HHVBP status during this earlier phase.

Nursing and therapy visits are the 2 most common services delivered by HHAs. In the postacute phase, skilled nursing services typically focus on clinical and medication management, while therapy visits help to restore or improve patients’ function. Regarding therapy services, prior research has shown that the provision and increased intensity of home health therapy services contribute to improved home health outcomes, including higher rates of successful community discharge and lower institutionalization rates, which correspond to several HHVBP performance metrics.^8,9,10,34,35^ Our finding of a higher number of therapy visits in HHVBP states in the first 14 and 30 days of home health may thus partly explain why previous studies have found that patients in HHVBP states experienced better overall outcomes than those in non-HHVBP states.^17^ Interestingly, the association between HHVBP and a higher number of therapy visits was observed in the context of PDGM reforms, which removed therapy thresholds and were expected to reduce the number of therapy visits. This increase in the number of therapy visits suggests that HHAs may be strategically emphasizing therapy services to improve outcomes and achieve favorable performance under HHVBP.

Regarding nursing services, while the number of nursing visits in 30 days was significantly lower in HHVBP states, no significant differences were observed during the first 14-day frontloading phase. Frontloading is a widely discussed concept in home health care that aims to increase visit intensity to achieve better outcomes. The temporal differences may suggest that HHAs prioritize clinical stabilization and caregiver education by maintaining nursing intensity in the early window, while increasing the number of therapy visits to restore and improve physical function over a relatively longer window (eg, 30 days). These findings likely reflect a service-mix strategy across different phases of the home health episode. A prior report also noted that HHAs considered that slowing the decrease in the number of nursing visits and accelerating the increase in the number of therapy visits could help achieve better quality scores.^36^ Lastly, while the effect size in our study was modest, possibly due to the observational window, the increase in therapy services was substantial from a population perspective. For example, with 68 765 residents in the HHVBP states, the increase of 0.32 therapy visits could equal more than 22 000 therapy visits compared with non-HHVBP states.

Consistent with prior research, we observed significant differences in home health visit frequency by patient race, ethnicity, and dual eligibility and HHA racial composition. The potential explanations for these differences by individual race, ethnicity, and socioeconomic status have been discussed elsewhere.^12,13,37,38,39^ Our study contributes additional insight by highlighting that HHA racial composition may partially explain these differences. Specifically, in non-HHVBP states, minority-serving HHAs and LNE HHAs, which are often proxies for very small agencies, were more likely to deliver nursing rather than therapy services. While the precise reasons for this pattern remain unclear, possible explanations include workforce-related challenges, such as a limited number of therapists.^40^ These constraints may be especially pronounced in minority-serving HHAs, which often operate with fewer resources and serve communities with lower socioeconomic status.^13,41^

The smaller differences associated with patient and HHA characteristics in HHVBP states represent a notable finding. Prior research has shown mixed impacts of other VBP programs on equity, with some studies suggesting an unintended widening of outcome differences.^42,43,44^ The observed shifts in visit patterns, particularly among minority-serving and LNE HHAs, which transitioned from predominantly nursing-based care to increased therapy use under HHVBP incentives, suggest that VBP mechanisms in HHAs may help reduce differences in service delivery. Further research is needed to better identify the mechanisms through which HHVBP influences HHA behavior and care provision. For example, qualitative studies investigating the perspectives of HHA administrators and clinicians in HHVBP states could provide valuable insights into the operational strategies and decision-making processes adopted in response to value-based incentives. Additionally, with the nationwide expansion of HHVBP and the continued influence of PDGM reforms, it will be important to assess whether similar changes in service delivery emerge across the broader US home health landscape.

### Limitations

This study has several limitations. First, as an observational analysis, the findings represent associations rather than causal relationships. Interpretations regarding the direct association of HHVBP programs with visit frequency should therefore be made with caution. Second, this study relied on Medicare claims, which do not include information on patient preferences or the availability of informal caregivers, both of which likely affect service use and visit frequency.^45^ However, the random selection of HHVBP states by CMS may reduce concerns about systematic differences in these unmeasured factors across states. Third, we did not have information on patients’ physical or cognitive functioning. Instead, we relied on hospitalization characteristics and chronic condition indicators as proxies to approximate clinical severity. Fourth, although we examined visit frequency, we were unable to assess the clinical content, appropriateness, or quality of the visits; frequency alone should not be interpreted as a direct measure of the value or effectiveness of care delivered. Lastly, our analysis was limited to Medicare FFS beneficiaries, and the results may not be generalizable to individuals enrolled in Medicare Advantage plans, who may have different service use patterns and care management approaches.

## Conclusions

In this retrospective cohort study of Medicare beneficiaries with ADRD, residence in an HHVBP state was associated with fewer nursing visits and more therapy visits in the first 30 days of postacute home health. Additionally, the disparity in receipt of therapy visits between Black and Hispanic (compared with White) beneficiaries, dual-eligible (compared with non dual-eligible) beneficiaries, and those receiving care from minority-serving (compared with predominantly White-serving) HHAs was smaller in HHVBP states than non-HHVBP states. The observed association of HHVBP with a reduction in disparities in therapy visit frequency is encouraging and suggests that VBP models could reduce differences in postacute care. As HHVBP is implemented nationwide, further research is needed to confirm these findings.

## Notes

Supplement 1. **eTable 1.** Comparison of Baseline Characteristics Between Included and Excluded Participants **eTable 2.** Full Results of Negative Binomial Regression Models for 30-Day Home Health Visits **eTable 3.** Sensitivity Analysis: 14-Day Home Health Visits **eTable 4.** Average Marginal Effects for 14-Day Outcomes Home Health Visits **eFigure 1.** Flowchart of the Cohort Creation **eFigure 2.** Adjusted 30-Day Home Health Visit Count by Medicare–Medicaid Dual-Eligibility Status and HHVBP Residence **eFigure 3.** Adjusted 14-Day Home Health Visit Counts by Explanatory Variables and HHVBP Residence

Supplement 2.
Data Sharing Statement
