# Supplementary material for: Home Health Value-Based Purchasing and Postacute Home Health Visits Among Older Adults With Dementia
Source: JAMA Netw Open. 2026 May 13;9(5):e2612232. doi: 10.1001/jamanetworkopen.2026.12232 (PMC13173378; doi:10.1001/jamanetworkopen.2026.12232)
Supplement: Supplement 1. — eTable 1. Comparison of Baseline Characteristics Between Included and Excluded Participants eTable 2. Full Results of Negative Binomial Regression Models for 30-Day Home Health Visits eTable 3. Sensitivity Analysis: 14-Day Home Health Visits eTable 4. Average Marginal Effects for 14-Day Outcomes Home Health Visits eFigure 1. Flowchart of the Cohort Creation eFigure 2. Adjusted 30-Day Home Health Visit Count by Medicare–Medicaid Dual-Eligibility Status and HHVBP Residence eFigure 3. Adjusted 14-Day Home Health Visit Counts by Explanatory Variables and HHVBP Residence [file jamanetwopen-e2612232-s001.pdf]

Supplemental Online Content

Yang M-T, Bloschichak A, Qin Q, Temkin-Greener H, Cai S. Home Health Value-Based Purchasing and visits among older adults with dementia. *JAMA Netw Open*. 2026;9(5):e2612232. doi:10.1001/jamanetworkopen.2026.12232

eTable 1. Comparison of Baseline Characteristics Between Included and Excluded Participants

eTable 2. Full Results of Negative Binomial Regression Models for 30-Day Home Health Visits

eTable 3. Sensitivity Analysis: 14-Day Home Health Visits

eTable 4. Average Marginal Effects for 14-Day Outcomes Home Health Visits

eFigure 1. Flowchart of the Cohort Creation

eFigure 2. Adjusted 30-Day Home Health Visit Count by Medicare–Medicaid Dual Eligibility Status and HHVBP Residence

eFigure 3. Adjusted 14-Day Home Health Visit Counts by Explanatory Variables and HHVBP Residence

This supplemental material has been provided by the authors to give readers additional information about their work.

eTable 1. Comparison of Baseline Characteristics Between Included and Excluded Participants

	Analytic cohort (N=264,601)	Excluded (N=356)	p-value
**Outcomes**			
No. of nursing visits*	4 (2 – 6)	4 (2 – 6)	0.16
No. of therapy visits*	6 (2 – 9)	6 (2 – 8)	0.28
**Explanatory variables**			
Race/ethnicity			
Non-Hispanic Whites	216,306 (81.7%)	241 (67.7%)	<0.001
Non-Hispanic Blacks	23,634 (8.9%)	63 (17.7%)	
Hispanics	16,562 (6.3%)	29 (8.1%)	
Asians	8,099 (3.1%)	23 (6.5%)	
Dual-eligibility status			
Non-duals	221,654 (83.8%)	297 (83.4%)	0.86
Duals	42,947 (16.2%)	59 (16.6%)	
HHA racial composition			
White-dominant HHA	194,780 (73.6%)	133 (37.4%)	<0.001
Minority-serving HHA	64,645 (24.4%)	154 (43.3%)	
LNE HHA	5,176 (2.0%)	69 (19.4%)	
**Individual-level covariates**			
Age*	83 (77 – 89)	85 (80 – 91)	<0.001
Sex			
Male	160,947 (60.8%)	207 (58.1%)	0.30
Female	103,654 (39.2%)	149 (41.9%)	
Total Medicare cost in the preceding 90 days of the index hospitalization ($)*	1,305 (393 – 3,393)	1018 (183 – 3,418)	
**Community-level covariates**			
Resided in deprived community			
No	251,229 (94.9%)	349 (98.0%)	0.008
Yes	13,372 (5.1%)	7 (2.0%)	
**Hospitalization-level covariates**			
DRG Weights*	1.31 (0.94 – 1.87)	1.43 (0.97 – 1.90)	0.20
LOS [a]	4 (2 – 6)	4 (3 – 7)	0.85
ICU			
No	191,349 (72.3%)	259 (72.8%)	0.85
Yes	73,252 (27.7%)	97 (27.2%)	
Hospitalization type			
Medical	210,066 (79.4%)	273 (76.7%)	0.21
Surgical	54,535 (20.6%)	83 (23.3%)	
**Chronic conditions**			

AMI	44,708 (16.9%)	59 (16.6%)	0.87
asthma	59,812 (22.6%)	78 (21.9%)	0.75
AF	116,654 (44.1%)	160 (44.9%)	0.75
Cancer	64,110 (24.2%)	79 (22.2%)	0.37
CKD	175,297 (66.2%)	218 (61.2%)	0.05
COPD	124,523 (47.1%)	151 (42.4%)	0.08
Depression	168,407 (63.6%)	162 (45.5%)	<0.001
DM	145,539 (55.0%)	191 (53.7%)	0.61
HF	156,552 (59.2%)	194 (54.5%)	0.07
Hip fracture	44,873 (17.0%)	70 (19.7%)	0.17
HLP	246,581 (93.2%)	317 (89.0%)	0.002
HTN	256,880 (97.1%)	337 (94.7%)	0.007
Ischemic heart disease	187,555 (70.9%)	229 (64.3%)	0.007
Osteoporosis	96,510 (36.5%)	122 (34.3%)	0.39
RA/OA	219,940 (83.1%)	275 (77.2%)	0.003
Stroke	129,277 (48.9%)	189 (53.1%)	0.11
**HHA covariates**			
5-star rating			
Quality >=3	216,980 (82.0%)	265 (74.4%)	<0.001
Quality <3 or no star	47,621 (18.0%)	91 (25.6%)	
Location			
Urban	235,715 (89.1%)	250 (70.2%)	<0.001
Rural	28,886 (10.9%)	106 (29.8%)	
Profit status			
For-profit	163,913 (61.9%)	250 (70.2%)	<0.001
Non-for-profit	95,049 (35.9%)	89 (25.0%)	
Governmental	5,639 (2.1%)	17 (4.8%)	
Operate branches			
No	158,709 (60.0%)	294 (82.6%)	<0.001
Yes	105,892 (40.0%)	62 (17.4%)	
HHA size			
Total bene < 60	6,815 (2.6%)	77 (21.6%)	<0.001
Total bene >= 60	257,786 (97.4%)	279 (78.4%)	

Variables with an asterisk indicate continuous variables, and we reported the median and included the interquartile range in parentheses. HHVBP: Home health value-based purchasing program; HHA: Home health agency; LNE: Low number of events; SNF/NH: Skilled nursing facility/nursing home; LOS: length of stay; ICU: Intensive care unit; AMI: Acute myocardial infarction; AF: Atrial fibrillation; CKD: Chronic kidney disease; COPD: Chronic obstructive pulmonary disease; DM: Diabetes mellitus; HF: Heart failure; HLP: Hyperlipidemia; HTN: Hypertension; RA/OA: Rheumatoid arthritis/Osteoarthritis

eTable 2. Full Results of Negative Binomial Regression Models for 30-day Home Health Visits

**Outcome**	**30-day nursing visits**				**30-day therapy visits**			
	Model 1	Model 2	Model 3	Model 4	Model 1	Model 2	Model 3	Model 4
**HHVBP status (Ref: non-HHVBP states)**								
HHVBP	-0.10***	-0.10***	-0.05***	-0.05***	0.06***	0.06***	0.03***	0.01**
	(-0.12 - -0.09)	(-0.11 - -0.09)	(-0.07 - -0.04)	(-0.06 - -0.03)	(0.05 - 0.07)	(0.05 - 0.07)	(0.02 - 0.04)	(0.00 - 0.03)
**Race/Ethnicity (Ref: non-Hispanic Whites) and interactions with HHVBP residence**								
Blacks	0.02***	0.00	-0.01	-0.01	-0.07***	-0.03***	-0.01**	-0.02**
	(0.01 - 0.03)	(-0.01 - 0.01)	(-0.02 - 0.00)	(-0.02 - 0.01)	(-0.08 - -0.05)	(-0.04 - -0.02)	(-0.03 - -0.00)	(-0.03 - -0.00)
Black x HHVBP	-0.01	-0.01	0.01	0.01	0.02	0.02	0.00	0.00
	(-0.04 - 0.01)	(-0.03 - 0.01)	(-0.02 - 0.03)	(-0.02 - 0.03)	(-0.00 - 0.05)	(-0.01 - 0.04)	(-0.02 - 0.03)	(-0.02 - 0.03)
Hispanics	0.03***	0.02***	0.01	0.01	-0.07***	-0.07***	-0.04***	-0.04***
	(0.02 - 0.05)	(0.01 - 0.03)	(-0.00 - 0.02)	(-0.00 - 0.02)	(-0.09 - -0.06)	(-0.08 - -0.05)	(-0.06 - -0.02)	(-0.06 - -0.02)
Hispanics x HHVBP	-0.03**	-0.03**	-0.01	-0.01	0.11***	0.10***	0.08***	0.07***
	(-0.06 - -0.00)	(-0.06 - -0.00)	(-0.03 - 0.02)	(-0.03 - 0.02)	(0.08 - 0.14)	(0.07 - 0.13)	(0.05 - 0.11)	(0.04 - 0.10)
Asians	-0.01	-0.03***	-0.04***	-0.04***	-0.03***	-0.03***	-0.01	-0.01
	(-0.03 - 0.00)	(-0.05 - -0.01)	(-0.06 - -0.02)	(-0.06 - -0.02)	(-0.05 - -0.01)	(-0.06 - -0.01)	(-0.03 - 0.01)	(-0.03 - 0.01)
Asians x HHVBP	-0.12***	-0.11***	-0.09***	-0.09***	0.05**	0.05	0.02	0.02
	(-0.17 - -0.07)	(-0.16 - -0.06)	(-0.14 - -0.04)	(-0.14 - -0.04)	(0.00 - 0.11)	(-0.00 - 0.10)	(-0.03 - 0.08)	(-0.03 - 0.08)
Duals	0.04***	0.03***	0.03***	0.03***	-0.13***	-0.11***	-0.10***	-0.10***
	(0.04 - 0.05)	(0.03 - 0.04)	(0.02 - 0.04)	(0.02 - 0.04)	(-0.14 - -0.12)	(-0.12 - -0.10)	(-0.11 - -0.09)	(-0.11 - -0.09)
Duals x HHVBP	-0.03***	-0.02**	-0.02	-0.02	0.04***	0.04***	0.03***	0.03***
	(-0.05 - -0.01)	(-0.04 - -0.00)	(-0.04 - 0.00)	(-0.04 - 0.00)	(0.02 - 0.06)	(0.02 - 0.06)	(0.01 - 0.05)	(0.01 - 0.05)
**HHA racial composition (Ref: White-dominant HHA) and interactions with HHVBP residence**								
Minority-serving HHA			0.11***	0.13***			-0.12***	-0.14***
			(0.09 - 0.13)	(0.11 - 0.15)			(-0.14 - -0.11)	(-0.15 - -0.12)
Minority-serving HHA x HHVBP			-0.22***	-0.23***			0.08***	0.10***
			(-0.26 - -0.19)	(-0.27 - -0.20)			(0.05 - 0.11)	(0.07 - 0.13)
LNE HHA			0.15***	0.12***			-0.35***	-0.30***
			(0.12 - 0.19)	(0.07 - 0.17)			(-0.38 - -0.31)	(-0.35 - -0.24)
LNE HHA x HHVBP			-0.15***	-0.16***			0.29***	0.30***
			(-0.22 - -0.08)	(-0.23 - -0.09)			(0.23 - 0.36)	(0.23 - 0.37)
**Individual-level covariates**								
Age (Ref: 65-74)								
75-84		-0.01	-0.00	-0.01		0.04***	0.04***	0.04***
		(-0.01 - 0.00)	(-0.01 - 0.00)	(-0.01 - 0.00)		(0.03 - 0.05)	(0.03 - 0.05)	(0.03 - 0.05)
>=85		-0.00	-0.00	-0.00		0.05***	0.05***	0.05***
		(-0.01 - 0.01)	(-0.01 - 0.01)	(-0.01 - 0.01)		(0.03 - 0.06)	(0.03 - 0.06)	(0.03 - 0.06)

Age	0.00***	0.00***	0.00***	0.01***	0.01***	0.01***
	(0.00 - 0.00)	(0.00 - 0.00)	(0.00 - 0.00)	(0.00 - 0.01)	(0.00 - 0.01)	(0.00 - 0.01)
Female (Ref: male)	0.01***	0.01**	0.01***	-0.00	0.00	0.00
	(0.00 - 0.01)	(0.00 - 0.01)	(0.00 - 0.01)	(-0.01 - 0.01)	(-0.01 - 0.01)	(-0.01 - 0.01)
Total Medicare cost in preceding 90 days of hospitalization (%)	-0.00***	-0.00***	-0.00***	-0.00***	-0.00***	-0.00***
	(-0.00 - -0.00)	(-0.00 - -0.00)	(-0.00 - -0.00)	(-0.00 - -0.00)	(-0.00 - -0.00)	(-0.00 - -0.00)
**Community-level covariates**						
Poor community (ADI>=85)	0.05***	0.05***	0.04***	-0.05***	-0.05***	-0.04***
	(0.03 - 0.06)	(0.03 - 0.06)	(0.03 - 0.05)	(-0.06 - -0.03)	(-0.06 - -0.03)	(-0.06 - -0.03)
COVID-19 cases per 1k 65+	-0.00	-0.00	-0.00	0.00	0.00	0.00
	(-0.00 - 0.00)	(-0.00 - 0.00)	(-0.00 - 0.00)	(-0.00 - 0.00)	(-0.00 - 0.00)	(-0.00 - 0.00)
**Hospitalization-level covariates**						
DRG weights	0.01***	0.01***	0.01***	-0.01***	-0.01***	-0.01***
	(0.01 - 0.02)	(0.01 - 0.02)	(0.01 - 0.02)	(-0.01 - -0.00)	(-0.01 - -0.00)	(-0.01 - -0.00)
LOS	0.01***	0.01***	0.01***	0.00***	0.00***	0.00***
	(0.01 - 0.01)	(0.01 - 0.01)	(0.01 - 0.01)	(0.00 - 0.00)	(0.00 - 0.00)	(0.00 - 0.00)
ICU utilization	0.02***	0.02***	0.02***	0.01***	0.01***	0.01***
	(0.02 - 0.03)	(0.02 - 0.03)	(0.02 - 0.03)	(0.01 - 0.02)	(0.01 - 0.02)	(0.01 - 0.02)
Surgical hospitalization (Ref: medical)	0.00	0.00	0.00	0.08***	0.08***	0.08***
	(-0.00 - 0.01)	(-0.00 - 0.01)	(-0.00 - 0.01)	(0.08 - 0.09)	(0.08 - 0.09)	(0.07 - 0.09)
**Chronic conditions**						
AMI	0.01**	0.01**	0.01**	-0.05***	-0.05***	-0.05***
	(0.00 - 0.02)	(0.00 - 0.02)	(0.00 - 0.01)	(-0.06 - -0.05)	(-0.06 - -0.05)	(-0.06 - -0.05)
Asthma	0.01**	0.01**	0.01**	0.01***	0.01***	0.01***
	(0.00 - 0.01)	(0.00 - 0.01)	(0.00 - 0.01)	(0.00 - 0.02)	(0.00 - 0.02)	(0.00 - 0.02)
AF	0.04***	0.04***	0.04***	-0.02***	-0.02***	-0.02***
	(0.03 - 0.04)	(0.03 - 0.04)	(0.03 - 0.04)	(-0.03 - -0.01)	(-0.03 - -0.01)	(-0.03 - -0.01)
Cancer	0.00	0.00	0.00	-0.03***	-0.03***	-0.03***
	(-0.00 - 0.01)	(-0.00 - 0.01)	(-0.00 - 0.01)	(-0.03 - -0.02)	(-0.03 - -0.02)	(-0.03 - -0.02)
CKD	0.03***	0.03***	0.03***	-0.04***	-0.04***	-0.04***
	(0.02 - 0.04)	(0.03 - 0.04)	(0.03 - 0.04)	(-0.04 - -0.03)	(-0.05 - -0.03)	(-0.05 - -0.03)
COPD	0.03***	0.03***	0.03***	-0.05***	-0.05***	-0.05***
	(0.02 - 0.04)	(0.02 - 0.04)	(0.02 - 0.04)	(-0.05 - -0.04)	(-0.05 - -0.04)	(-0.05 - -0.04)
Depression	-0.02***	-0.02***	-0.02***	0.03***	0.03***	0.03***
	(-0.02 - -0.01)	(-0.02 - -0.01)	(-0.02 - -0.01)	(0.02 - 0.04)	(0.02 - 0.04)	(0.02 - 0.04)
DM	0.05***	0.05***	0.05***	-0.01***	-0.01***	-0.01***

		(0.04 - 0.06)	(0.04 - 0.05)	(0.04 - 0.05)		(-0.02 - -0.01)	(-0.02 - -0.00)	(-0.02 - -0.01)
HF		0.07***	0.07***	0.06***		-0.04***	-0.04***	-0.04***
		(0.06 - 0.07)	(0.06 - 0.07)	(0.06 - 0.07)		(-0.05 - -0.04)	(-0.05 - -0.04)	(-0.05 - -0.03)
Hip fracture		-0.03***	-0.03***	-0.03***		0.11***	0.11***	0.11***
		(-0.04 - -0.03)	(-0.04 - -0.03)	(-0.04 - -0.03)		(0.10 - 0.12)	(0.10 - 0.12)	(0.10 - 0.12)
Hyperlipidemia		-0.00	-0.00	-0.00		0.03***	0.03***	0.03***
		(-0.02 - 0.01)	(-0.02 - 0.01)	(-0.01 - 0.01)		(0.02 - 0.04)	(0.02 - 0.04)	(0.02 - 0.04)
Hypertension		0.06***	0.06***	0.06***		0.02	0.02**	0.02
		(0.04 - 0.08)	(0.04 - 0.08)	(0.04 - 0.08)		(-0.00 - 0.03)	(0.00 - 0.04)	(-0.00 - 0.03)
Ischemic heart disease		0.03***	0.03***	0.03***		-0.02***	-0.02***	-0.02***
		(0.03 - 0.04)	(0.03 - 0.04)	(0.03 - 0.04)		(-0.03 - -0.01)	(-0.03 - -0.01)	(-0.03 - -0.01)
Osteoporosis		0.00	0.00	0.00		0.02***	0.03***	0.03***
		(-0.00 - 0.01)	(-0.00 - 0.01)	(-0.00 - 0.01)		(0.02 - 0.03)	(0.02 - 0.03)	(0.02 - 0.03)
RA/OA		-0.02***	-0.02***	-0.02***		0.08***	0.08***	0.08***
		(-0.03 - -0.02)	(-0.03 - -0.02)	(-0.03 - -0.02)		(0.07 - 0.09)	(0.07 - 0.09)	(0.07 - 0.09)
Stroke		-0.04***	-0.04***	-0.04***		0.07***	0.07***	0.07***
		(-0.05 - -0.04)	(-0.05 - -0.04)	(-0.05 - -0.04)		(0.07 - 0.08)	(0.07 - 0.08)	(0.07 - 0.08)
**HHA-level covariates**								
Low quality HHA				0.00				-0.08***
				(-0.01 - 0.02)				(-0.09 - -0.07)
Located in rural areas				0.16***				-0.06***
				(0.14 - 0.18)				(-0.08 - -0.05)
Profit status (Ref: for profit)								
Non-for-profit				0.02**				-0.11***
				(0.00 - 0.04)				(-0.12 - -0.09)
Government				-0.04**				0.04**
				(-0.08 - -0.00)				(0.01 - 0.08)
Operates branch				-0.04***				0.02***
				(-0.05 - -0.02)				(0.01 - 0.03)
Beneficiary >=60				-0.07***				0.07***
				(-0.11 - -0.03)				(0.03 - 0.11)
# of obs	264,601	264,601	264,601	264,601	264,601	264,601	264,601	264,601
# of HHA	7,825	7,825	7,825	7,825	7,825	7,825	7,825	7,825

Values are presented as estimated beta coefficients from the negative binomial regression models, with 95% CIs reported in parentheses. “x” means interactions. Model 1 was an unadjusted model including only explanatory variables and their interaction terms; Model 2 further adjusted for individual characteristics (eg, age, sex, chronic conditions), index hospitalization characteristics (eg, DRG weights and LOS), and community-level covariates (e.g., residence in a deprived community). The total Medicare cost in the preceding 90 days was log-transformed; Model 3 added HHA racial composition and its interaction with HHVBP status; Model 4 was the fully adjusted model, including other HHA-level covariates. All models included HHA random effects to account for patient clustering. HHVBP: home health value-based purchasing; LNE: low number of events; HHA: home health agency; ADI: Area Deprivation Index; DRG: diagnosis-related group; LOS: length of stay; ICU: intensive care unit; AMI: acute myocardial infarction; AF: atrial

fibrillation; CKD: chronic kidney disease; COPD: chronic obstructive pulmonary disease; DM: diabetes mellitus; HF: heart failure; RA/OA: rheumatoid arthritis and osteoarthritis; obs: observations.*** $p<0.01$, ** $p<0.05$.

eTable 3. Sensitivity analysis: 14-day Home Health Visits

	14-day outcome	
	Nursing visits	Therapy visits
**HHVBP status (Ref: non-HHVBP states)**		
HHVBP	0.00	0.02**
	(-0.01 - 0.02)	(0.00 - 0.03)
**Race/Ethnicity (Ref: non-Hispanic Whites)**		
Blacks	-0.02***	-0.04***
	(-0.03 - -0.01)	(-0.06 - -0.03)
x HHVBP	-0.00	0.00
	(-0.02 - 0.02)	(-0.02 - 0.03)
Hispanics	0.00	-0.05***
	(-0.01 - 0.02)	(-0.06 - -0.03)
x HHVBP	0.03**	0.06***
	(0.00 - 0.05)	(0.03 - 0.09)
Asians	-0.03***	0.00
	(-0.05 - -0.02)	(-0.02 - 0.02)
x HHVBP	-0.06**	0.01
	(-0.11 - -0.01)	(-0.04 - 0.06)
Duals	0.02***	-0.08***
	(0.01 - 0.03)	(-0.09 - -0.07)
x HHVBP	0.00	0.03***
	(-0.02 - 0.02)	(0.01 - 0.05)
**HHA racial composition (Ref: White-dominant HHA)**		
Minority-serving	0.07***	-0.18***
	(0.05 - 0.09)	(-0.20 - -0.16)
x HHVBP	0.03	0.11***
	(-0.00 - 0.07)	(0.08 - 0.14)
LNE HHA	0.13***	-0.31***
	(0.09 - 0.18)	(-0.36 - -0.25)
x HHVBP	-0.04	0.28***
	(-0.10 - 0.03)	(0.21 - 0.35)
**Individual-level covariates**		
Age (Ref: 65-74)		
75-84	-0.01**	0.02***
	(-0.02 - -0.00)	(0.02 - 0.03)
>=85	-0.00	0.03***
	(-0.02 - 0.01)	(0.01 - 0.04)
Age	0.00***	0.00***
	(0.00 - 0.00)	(0.00 - 0.01)
Female (Ref: male)	0.01***	0.01**
	(0.01 - 0.02)	(0.00 - 0.01)
Total Medicare cost in preceding 90 days of hospitalization (%)	-0.00***	-0.01***
	(-0.00 - -0.00)	(-0.01 - -0.00)
Poor community (ADI>=85)	0.03***	-0.04***
	(0.02 - 0.04)	(-0.06 - -0.03)
COVID-19 cases per 1k 65+	-0.00	-0.00
	(-0.00 - 0.00)	(-0.00 - 0.00)
**Hospitalization-level covariates**		
DRG weights	0.01***	-0.01***
	(0.01 - 0.01)	(-0.01 - -0.01)
LOS	0.01***	0.00***
	(0.01 - 0.01)	(0.00 - 0.00)
ICU utilization	0.01***	0.01**
	(0.00 - 0.02)	(0.00 - 0.01)
Surgical hospitalization (Ref: medical)	0.04***	0.12***

	(0.03 - 0.05)	(0.11 - 0.13)
**Chronic conditions**		
AMI	0.00	-0.05***
	(-0.01 - 0.01)	(-0.06 - -0.04)
Asthma	0.00	0.00
	(-0.00 - 0.01)	(-0.00 - 0.01)
AF	0.03***	-0.02***
	(0.02 - 0.03)	(-0.03 - -0.02)
Cancer	0.00	-0.02***
	(-0.00 - 0.01)	(-0.03 - -0.02)
CKD	0.03***	-0.04***
	(0.02 - 0.03)	(-0.04 - -0.03)
COPD	0.02***	-0.04***
	(0.02 - 0.03)	(-0.05 - -0.04)
Depression	-0.01***	0.02***
	(-0.02 - -0.01)	(0.01 - 0.02)
DM	0.04***	-0.02***
	(0.03 - 0.04)	(-0.02 - -0.01)
HF	0.05***	-0.05***
	(0.04 - 0.05)	(-0.05 - -0.04)
Hip fracture	-0.03***	0.10***
	(-0.03 - -0.02)	(0.09 - 0.10)
Hyperlipidemia	0.00	0.03***
	(-0.01 - 0.01)	(0.02 - 0.04)
Hypertension	0.04***	-0.00
	(0.02 - 0.05)	(-0.02 - 0.02)
Ischemic heart disease	0.02***	-0.02***
	(0.02 - 0.03)	(-0.02 - -0.01)
Osteoporosis	0.00	0.02***
	(-0.00 - 0.01)	(0.01 - 0.02)
RA/OA	-0.02***	0.07***
	(-0.03 - -0.01)	(0.06 - 0.07)
Stroke	0.00	-0.05***
	(-0.01 - 0.01)	(-0.06 - -0.04)
HHA-level covariates		
Low quality HHA	-0.04***	0.06***
	(-0.05 - -0.04)	(0.06 - 0.07)
Located in rural areas	0.06***	-0.09***
	(0.04 - 0.08)	(-0.10 - -0.07)
Profit status (Ref: for profit)		
Non-for-profit	-0.01	-0.06***
	(-0.03 - 0.01)	(-0.08 - -0.04)
Government	-0.02	0.10***
	(-0.06 - 0.02)	(0.06 - 0.14)
Operates branch	-0.07***	0.00
	(-0.09 - -0.05)	(-0.02 - 0.02)
Beneficiary >=60	-0.02	0.06**
	(-0.06 - 0.02)	(0.01 - 0.10)
# of obs	264,601	264,601
# of HHA	7,825	7,825

Values are presented as estimated beta coefficients from the fully-adjusted negative binomial regression models, with 95% CIs reported in parentheses. "x" means interaction. All models included HHA random effects to account for patient clustering. HHVBP: home health value-based purchasing; LNE: low number of events; HHA: home health agency; ADI: Area Deprivation Index; DRG: diagnosis-related group; LOS: length of stay; ICU: intensive care unit;.*** p<0.01, ** p<0.05.

eTable 4. Average Marginal Effects for 14-day Home Health Visits

	**14-day nursing visits**			**14-day therapy visits**		
	Non-HHVBP	HHVBP	p-value	Non-HHVBP	HHVBP	p-value
**Race/Ethnicity (Ref: non-Hispanic Whites)**						
Blacks	-0.05***	-0.06**	0.90	-0.14***	-0.14***	0.96
	(-0.08 – 0.03)	(-0.11 – -0.01)		(-0.18 – -0.10)	(-0.20 – 0.07)	
Hispanics	0.01	0.08***	0.04	-0.16***	0.03	<0.001
	(-0.02 – 0.04)	(0.02 – 0.14)		(-0.20 – -0.11)	(-0.06 – 0.12)	
Asians	-0.08***	-0.24***	0.01	0.00	0.03	0.76
	(-0.13 – -0.04)	(-0.34 – -0.13)		(-0.06 – 0.07)	(-0.12 – 0.19)	
**Dual eligibility (Ref: non-dual)**						
Duals	0.06***	0.06***	0.86	-0.26***	-0.16***	0.005
	(0.04 – 0.18)	(0.02 – 0.11)		(-0.29 – -0.23)	(-0.22 – -0.10)	
**HHA racial composition (Ref: White-dominant HHA)**						
Minority serving HHA	0.20***	0.29 ***	0.06	-0.57***	-0.24***	<0.001
	(0.14 – 0.25)	(0.20 – 0.39)		(-0.63 – -0.52)	(-0.35 – -0.14)	
LNE HHA	0.37***	0.26***	0.25	-0.91***	-0.09***	<0.001
	(0.23 – 0.52)	(0.07 – 0.46)		(-1.04 – -0.77)	(-0.35 – 0.16)	

The values shown in this table were the average marginal effects estimated from the fully adjusted negative binomial regression model (Model 4), with 95% CIs reported in parentheses. The interpretation of the values is the adjusted differences in the 30-day nursing or therapy visits between groups and their counterparts. The p-values were calculated using the pwcompare(effects) command in STATA syntax to test whether the two estimated average marginal effects were statistically significantly different. HHVBP: Home health value-based purchasing program; HHA: Home health agency; LNE: low number of events *** p<0.01, ** p<0.05.

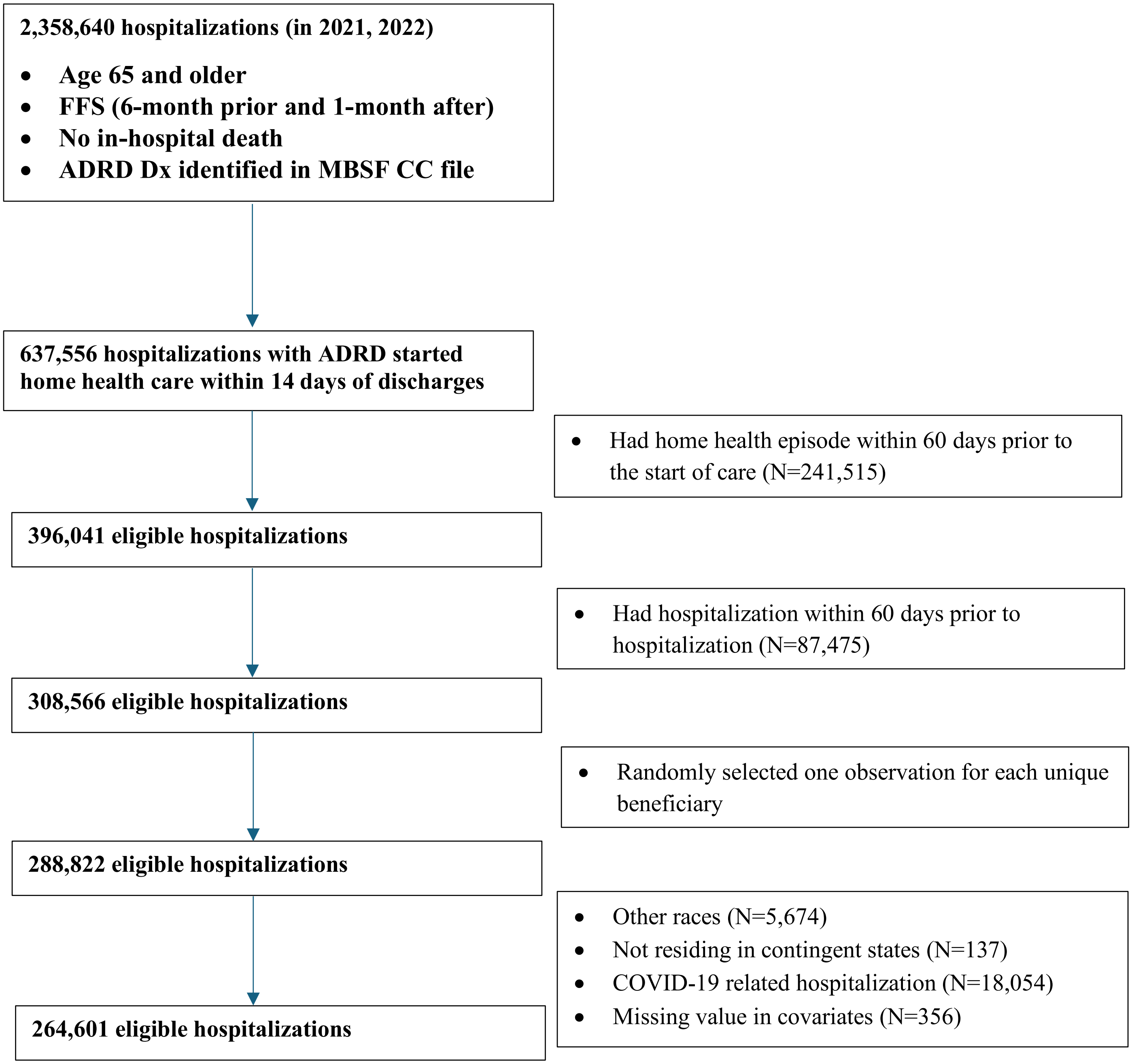

eFigure 1. Flowchart of the cohort creation

FFS: fee-for-services; ADRD: Alzheimer's Disease and Related Dementias; Dx: diagnosis; MBSF: Master Beneficiary Summary File; CC: Chronic condition.

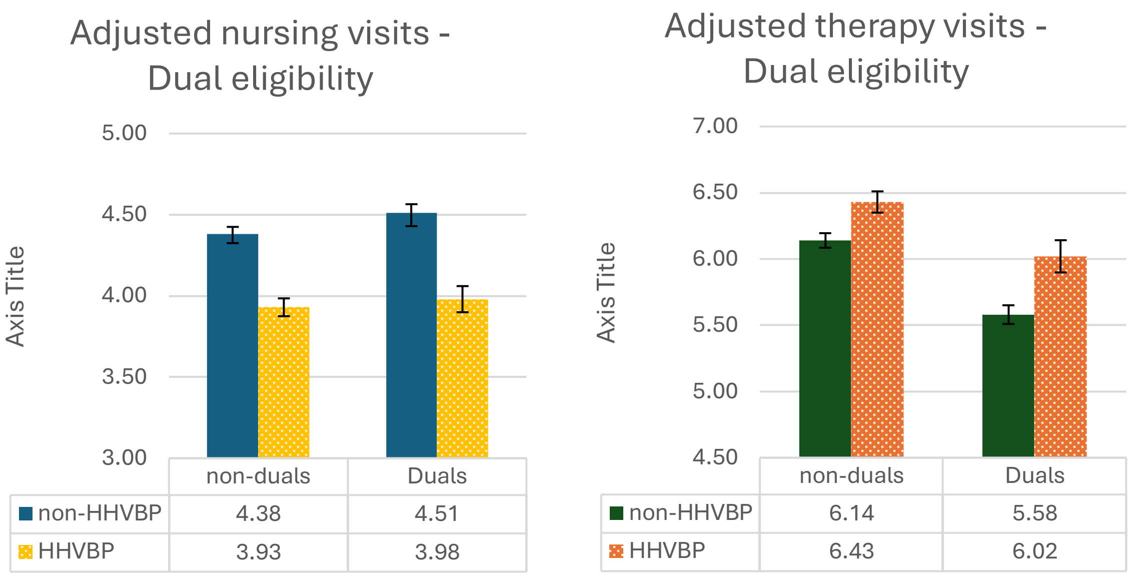

eFigure 2. Adjusted 30-day Home Health Visit Counts by Medicare-Medicaid Dual Eligibility Status and HHVBP residence

The values shown in this table were the adjusted counts estimated from the fully adjusted negative binomial regression model (Model 4). HHVBP: Home health value-based purchasing program; HHA: Home health agency; LNE: low number of events.

© 2026 Yang M-T, et al. *JAMA Network Open*.

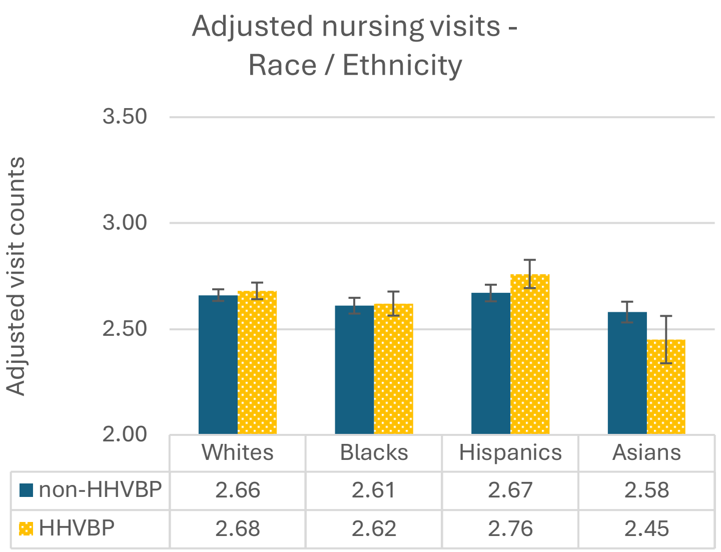

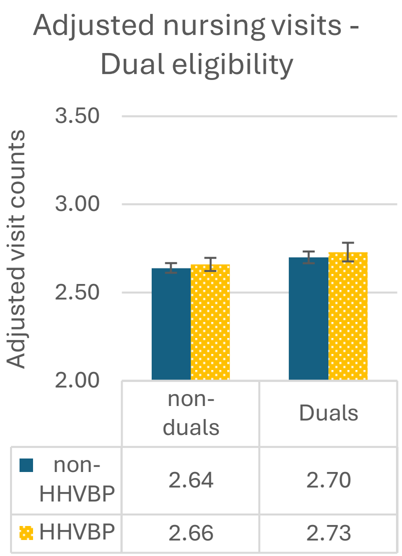

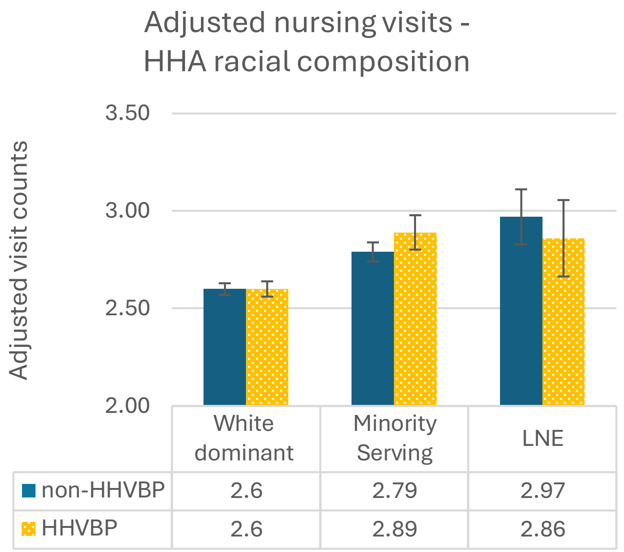

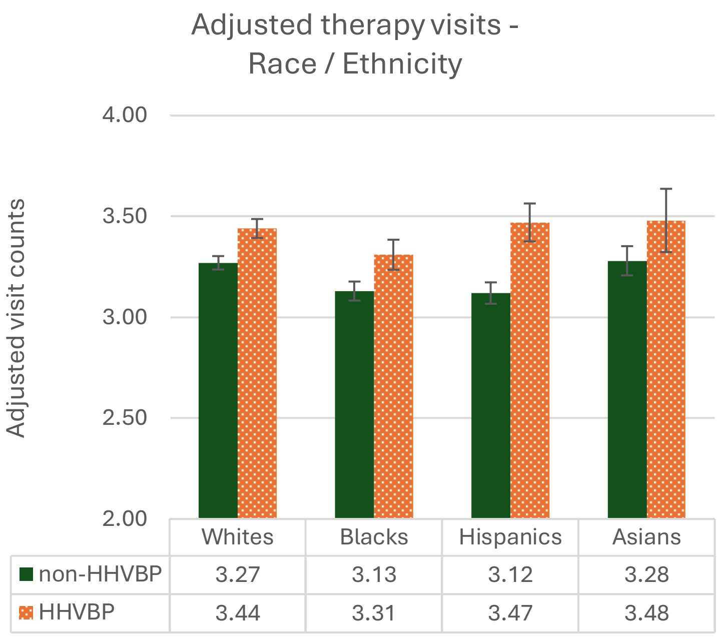

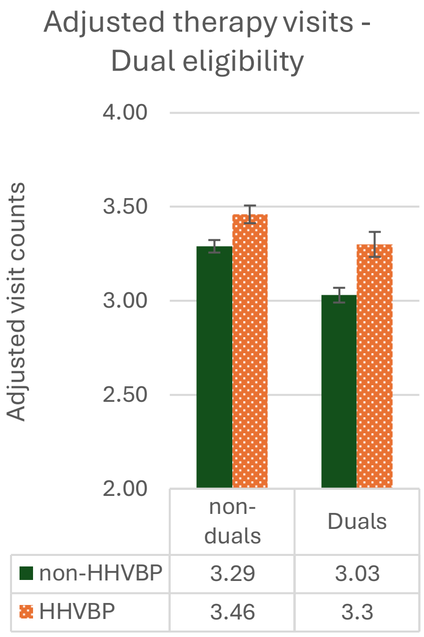

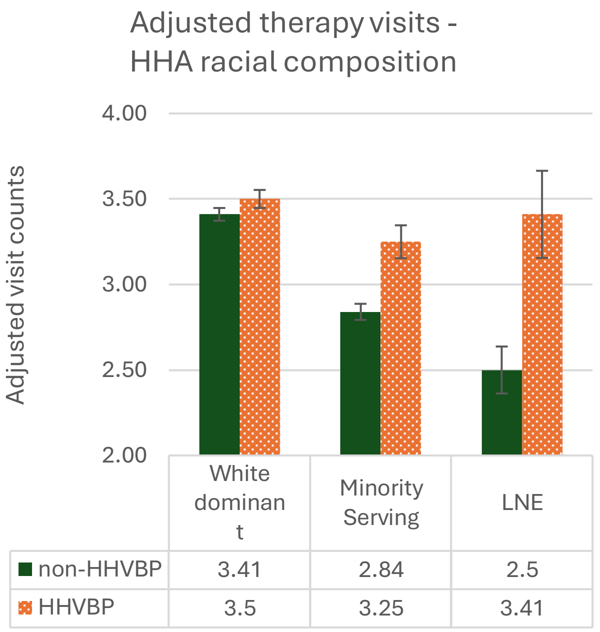

© 2026 Yang M-T, et al. *JAMA Network Open*.

eFigure 3. Adjusted 14-day Home Health Visit Counts by Explanatory Variables and HHVBP residence

The values shown in this table were the adjusted counts estimated from the fully adjusted negative binomial regression model. HHVBP: Home health value-based purchasing program; HHA: Home health agency; LNE: low number of events.

© 2026 Yang M-T, et al. *JAMA Network Open*.
