# Supplementary material for: Home Health Value-Based Purchasing and Postacute Home Health Visits Among Older Adults With Dementia
Source: JAMA Netw Open. 2026 May 13;9(5):e2612232. doi: 10.1001/jamanetworkopen.2026.12232 (PMC13173378; doi:10.1001/jamanetworkopen.2026.12232)
Supplement: Supplement 2. — Data Sharing Statement [file jamanetwopen-e2612232-s002.pdf]

Data Sharing Statement

Yang. Home Health Value-Based Purchasing and Postacute Home Health Visits Among Older Adults With Dementia. *JAMA Netw Open*. Published May 13, 2026.
doi:10.1001/jamanetworkopen.2026.12232

Data
Data available: No
